# Supplementary material for: Evaluation of a screening algorithm using the Strengths and Difficulties Questionnaire to identify children with mental health problems: A five-year register-based follow-up on school performance and healthcare use
Source: PLoS One. 2019 Oct 23;14(10):e0223314. doi: 10.1371/journal.pone.0223314 (PMC6808306; doi:10.1371/journal.pone.0223314)
Supplement: S1 Table — (DOCX) [file pone.0223314.s001.docx]

**S1 Table**

**Table A: The association between mental health problems at age 11-12 years and not completing the 9^th^ grade exams**

|  | Crude model  N=2,015 | Model including child’s characteristics  N=2,015 | Model including child’s characteristics and IQ-proxy  N=1,537 | Model including child’s and parent’s characteristics N=2,015 | Model including child’s, parent’s characteristics, and IQ-proxy  N=1,537 |
| --- | --- | --- | --- | --- | --- |
| Mental health problems | 3.22 (0.91) *** | 3.14 (0.91) *** | 1.71 (0.68) | 3.00 (0.87)*** | 1.57 (0.65) |
| Girl |  | 0.53 (9.13) ** | 0.53 (0.16)** | 0.54 (0.14)** | 0.51 (0.15)** |
| First-born in the family |  | 1.04 (0.25) | 1.09 (0.31) | 1.01 (0.25) | 1.04 (0.31) |
| Born with low-birth-weight or small-for-gestational-age |  | 0.39 (0.29) | 0.27 (0.27) | 0.34 (0.25) | 0.24 (0.24) |
| Parents are first- or second-generation immigrants |  | 1.84 (0.59)* | 1.56 (0.62) | 1.60 (0.60) | 1.34 (0.62) |
| Child did not live with both biological parents 1. Jan 2012 |  | 1.23 (0.33) | 1.31 (0.42) | 1.05 (0.60) | 1.11 (0.37) |
| Mother had contact to the mental health hospital services between 1995 and 2011 |  |  |  | 1.74 (0.63) | 2.28 (1.01)* |
| Father had contact to the mental health hospital services between 1995 and 2011 |  |  |  | 0.69 (0.36) | 0.91 (0.53) |
| Mother’s highest education primary school (up to 9 years) |  |  |  | 0.51 (0.20)* | 0.60 (0.27) |
| Father’s highest education primary school (up to 9 years) |  |  |  | 1.66 (0.50)* | 1.05 (0.41) |
| Mother was unemployed in 2011 |  |  |  | 1.08 (0.50) | 1.72 (0.87) |
| Father was unemployed in 2011 |  |  |  | 2.02 (0.86)* | 1.95 (0.98) |
| Mother among the 25% of the CCC2000 mothers with highest health care costs between 2000 and 2011 |  |  |  | 1.37 (0.38) | 0.99 (0.36) |
| Father among the 25% of the CCC2000 fathers with highest health care costs between 2000 and 2011 |  |  |  | 1.59 (0.42)* | 1.38 (0.44) |
| Block Design Score |  |  | 0.91 (0.03)*** |  | 0.91 (0.03)** |

Note: The table contains estimated Odds Ratios (OR) of having a mental health problem at age 11-12 years and not completing the 9^th^ grade exams. Logistic regression models are used to estimate OR with standard error (SE). As not all children have an IQ-test score the model including this population has fewer observations. We define not having completed the 9^th^ grade exams as not attending at least four of the eight mandatory exams.

*p-level <0.1 **p-level <0.05 ***p-level <0.01.

**Table B: The association between mental health problems at age 11-12 years and Grade Point Average at 9th grade exams**

|  | Crude model  N=1,941 | Model including child’s characteristics  N=1,941 | Model including child’s characteristics. and IQ-proxy  N=1,486 | Model including child’s and parent’s characteristics N=1,941 | Model including child’s, parent’s characteristics, and IQ-proxy  N=1,486 |
| --- | --- | --- | --- | --- | --- |
|  | ME (SE) | | | | |
| Mental health problems | -1.27 (0.19) *** | -1.13 (0.18)*** | -1.03 (0.19)*** | -1.04 (0.18)*** | -0.98 (0.19)*** |
| Girl |  | 0.80 (0.10)*** | 0.67 (0.10)*** | 0.75 (0.10)*** | 0.63 (0.10)*** |
| First-born in the family |  | 0.09 (0.10) | 0.23 (0.10)** | 0.11 (0.10) | 0.23 (0.10)** |
| Born with low-birth-weight or small-for-gestational-age |  | -0.61 (0.20)*** | -0.30 (0.22) | -0.48 (0.20)** | -0.24 (0.23) |
| Parents are first- or second-generation immigrants |  | -2.20 (0.17)*** | -1.59 (0.19)*** | -1.19 (0.19)*** | -0.84 (0.21)*** |
| Child did not live with both biological parents 1. Jan 2012 |  | -0.86 (0.11)*** | -0.64 (0.13)*** | -0.58 (0.12)*** | -0.41 (0.13)*** |
| Mother had contact to the mental health hospital services between 1995 and 2011 |  |  |  | 0.22 (0.19) | 0.24 (0.22) |
| Father had contact to the mental health hospital services between 1995 and 2011 |  |  |  | -0.27 (0.23) | -0.30 (0.24) |
| Mother’s highest education primary school (up to 9 years) |  |  |  | -0.88 (0.16)*** | -0.69 (0.19)*** |
| Father’s highest education primary school (up to 9 years) |  |  |  | -0.91 (0.15)*** | -0.71 (0.16)*** |
| Mother was unemployed in 2011 |  |  |  | -1.01 (0.22)*** | -0.76 (0.26)*** |
| Father was unemployed in 2011 |  |  |  | -0.42 (0.24)* | -0.40 (0.27) |
| Mother among the 25% of the CCC2000 mothers with highest health care costs between 2000 and 2011 |  |  |  | 0.11 (0.13) | 0.12 (0.14) |
| Father among the 25% of the CCC2000 fathers with highest health care costs between 2000 and 2011 |  |  |  | -0.17(0.12) | -0.27 (0.12)** |
| Block Design Score |  |  | 0.22 (0.01)*** |  | 0.20 (0.01)*** |

Note: Note: The table contains estimated coefficients (in grade points) for the association between mental health problems at age 11-12 years and Grade Point Average (GPA) at 9^th^ grade exams. The study population includes children who have done at least four of the 9^th^ grade exams. As not all children have an IQ-test score the model including this population has fewer observations. Ordinary Least Square regression models are used to estimate Marginal effect (ME) with robust standard error (SE) of mental health problems on GPA. *p-level <0.1 **p-level <0.05 ***p-level <0.01.

**Table C: The associations between mental health problems at age 11-12 years and total health care costs in the period 2012 to 2016**

|  | Crude model | Model including child’s characteristics | Model including child’s and parent’s characteristics |
| --- | --- | --- | --- |
|  | ME (SE) | | |
| Mental health problems | 6,044 (2,254)*** | 7,336 (2,301)*** | 5,858 (1,713)*** |
| Girl |  | 2,436 (1,122)** | 2,232 (1,142)* |
| First-born in the family |  | 1,711 (1,111) | 1,419 (1,142) |
| Born with low-birth-weight or small-for-gestational-age |  | -2,121 (817)*** | -1.946 (765)** |
| Parents are first- or second-generation immigrants |  | -1,845 (751)** | -1,810 (763)** |
| Child did not live with both biological parents 1. Jan 2012 |  | 1,340 (1,582) | 1,259 (1,782) |
| Mother had contact to the mental health hospital services between 1995 and 2011 |  |  | 1,248 (1,566) |
| Father had contact to the mental health hospital services between 1995 and 2011 |  |  | 313 (1,127) |
| Mother’s highest education primary school (up to 9 years) |  |  | -441 (987) |
| Father’s highest education primary school (up to 9 years) |  |  | 2,469 (1,328)* |
| Mother was unemployed in 2011 |  |  | -1.852 (723)** |
| Father was unemployed in 2011 |  |  | -2,158 (721)*** |
| Mother among the 25% of the CCC2000 mothers with highest health care costs between 2000 and 2011 |  |  | 1,676 (766)** |
| Father among the 25% of the CCC2000 fathers with highest health care costs between 2000 and 2011 |  |  | 1,441 (1000) |

Note: The table contains estimated coefficients (in €) of the association between mental health problems at age 11-12 years and total health care costs in the following five year-period. The number of observations N=2,015. Marginal effect (ME) and robust standard error (SE) estimated with a GLM model.

*p-level <0.1 **p-level <0.05 ***p-level <0.01.

**Table D: The associations between mental health problems at age 11-12 years with/without specialised mental health care and health care cost in the period 2012 to 2016**

|  | Crude models | | Model including child’s characteristics^c^ | | Model including child’s and parent’s characteristics^c,d^ | |
| --- | --- | --- | --- | --- | --- | --- |
|  | Mental health problems and no specialised mental health care (n=150) | Mental health problems and specialised mental health care (n=44) | Mental health problems and no specialised mental health care (n=150) | Mental health problems and specialised mental health care (n=44) | Mental health problems and no specialised mental health care (n=150) | Mental health problems and specialised mental health care (n=44) |
|  | ME (SE) | ME (SE) | ME (SE) | ME (SE) | ME (SE) | ME (SE) |
| Total health care costs^a^ | -918 (930) | 29,778 (8,325)*** | -90 (960) | 32,568 (8,706)*** | -71 (996) | 27,781 (6,832)*** |
| Primary sector care without psychologist/psychiatrist^a^ | 126 (84) | 365 (139)*** | 118 (78) | 373 (160)** | 99 (78) | 322 (158)** |
| Psychologist/psychiatrist in primary care sector^b^ | NA | 1,004 (435)** | NA | 769 (262)*** | NA | 1,023 (522)* |
| Prescription medicine^b^ | -18 (62) | 775 (244)*** | -20 (60) | 714 (250)*** | -18 (51) | 879 (330)*** |
| Mental outpatient hospital care^b^ | NA | 3,853 (727)*** | NA | 3,943 (769)*** | NA | 3,853 (822)*** |
| Mental inpatient hospital care^b^ | NA | 22,043 (7,917)*** | NA | 22,592 (7,613)*** | NA | 57,833 (67,081) |
| Somatic outpatient hospital care^b^ | 157 (292) | 929 (508)* | 151 (275) | 913 (502)* | 37 (240) | 685 (425) |
| Somatic inpatient hospital care^b^ | -166 (673) | 809 (828) | 545 (818) | 1,210 (948) | 723 (856) | 1,570 (1,079) |

Note: The table contains estimated coefficients (in €) of the association between mental health problems at age 11-12 years and health care costs in the following five year-period. The number of observations N=2,015. Some estimates are non-applicable (NA) based on the definition of the group. Specialised mental health care (SMHC) is defined as contact with publicly funded psychiatrist or psychologist.

^a^ Marginal effect (ME) and robust standard error (SE) estimated with a GLM model.

^b^ Marginal effect (ME) and robust standard error (SE) estimated with a two-part model combining a logit and a GLM model.

^c^ Gender, first-born child in the family, born small-for-gestational-age or with low-birth-weight, parents are second generation immigrants, child did not live with both biological parents 1st January 2012.

^d^ Mother and/or father had contact to mental health hospital services between 1995 and 2011, mother’s and/or father’s highest education on 1st January 2012 was primary school (up to 9 years schooling), mother and/or father was unemployed in 2011, mother and/or father was among the parents of the full CCC2000 cohort with the 25% highest health care cost between 2000 and 2011.

*p-level <0.1 **p-level <0.05 ***p-level <0.01.

**Table E: The full Copenhagen Child Cohort (CCC2000) population and the population of children with Strengths and Difficulties questionnaire (SDQ) data at age 11-12 years**

|  | The full CCC2000 population | Children with SDQ data at age 11-12 years |
| --- | --- | --- |
| Girl | 48.7% | 50.3% * |
| First-born in the family | 51.8% | 50.4% |
| Born with low-birth-weight or small-for-gestational-age | 6.5% | 6.1% |
| Parents are first- or second-generation immigrants | 16.4% | 10.4% *** |
| Child did not live with both biological parents 1. Jan 2012 | 21.5% | 14.5% *** |
| Mother had contact to the mental health hospital services between 1995 and 2011 | 7.8% | 6.7% ** |
| Father had contact to the mental health hospital services between 1995 and 2011 | 4.2% | 3.4% ** |
| Mother’s highest education primary school (up to 9 years) | 23.2% | 14.1% *** |
| Father’s highest education primary school (up to 9 years) | 25.2% | 17.5% *** |
| Mother was unemployed in 2011 | 17.2% | 9.6% *** |
| Father was unemployed in 2011 | 7.6% | 4.4% *** |
| Mother among the 25 % with highest health care sector costs between 2000 and 2011 in the study population | 25.0% | 23.2% ** |
| Father among the 25 % with highest health care sector costs between 2000 and 2011 in the study population | 25.0% | 24.7% |
| N | 6,090 | 2,126 |

Note: The table describes the characteristics of children and their parents in the Copenhagen Child Cohort by whether they are part of the study population or not. Differences in the characteristics across the children’s mental problem status is tested using a Chi-square test. *p-level <0.1 **p-level <0.05 ***p-level <0.01.

**Table F: The association between mental health problems at age 5-7 and Grade Point Average at 9th grade exams**

|  | Mental health problems at age 5-7 years | |
| --- | --- | --- |
|  | ME | SE |
| Crude model | -0.57*** | 0.21 |
| Model including child’s characteristics^a^ | -0.25 | 0.21 |
| Model including child’s and parent’s characteristics^a,b^ | -0.17 | 0.19 |

Note: The table contains estimated coefficients (in grade points) for the association between mental health problems at age 11-12 years and Grade Point Average (GPA) at 9^th^ grade exams. N=3,105. The study population includes children who have done at least four of the 9^th^ grade exams. Ordinary Least Square regression models are used to estimate Marginal effect (ME) with robust standard error (SE) of mental health problems on GPA

^a^ Gender, first-born child in the family, born small-for-gestational-age or with low-birth-weight, parents are second generation immigrants, child did not live with both biological parents 1st January 2006.

^b^ Mother and/or father had contact to mental health hospital services between 1995 and 2005, mother’s and/or father’s highest education on 1st January 2006 was primary school (up to 9 years schooling), mother and/or father was unemployed in 2005, mother and/or father was among the parents of the full CCC2000 cohort with the 25% highest health care cost between 2000 and 2005.

*p-level <0.1 **p-level <0.05 ***p-level <0.01.

**Table G: The associations between mental health problems at age 5-7 and health care costs in the period 2006 to 2016**

|  | Crude models | | Models including child’s characteristics^c^ | | Models including child’s and parent’s characteristics^c,d^ | |
| --- | --- | --- | --- | --- | --- | --- |
|  | Mental health problems at age 5-7 years | | | | | |
|  | ME | SE | ME | SE | ME | SE |
| Total health care costs^a^ | 5,155 ** | 2,120 | 4,885 ** | 1,958 | 4,338 ** | 1,691 |
| Primary sector care without psychologist/psychiatrist^a^ | 615 * | 329 | 619* | 334 | 467 ** | 191 |
| Psychologist/psychiatrist in primary care sector^a^ | 277 ** | 156 | 257 ** | 111 | 339 ** | 155 |
| Prescription medicine^b^ | 2,183 *** | 543 | 2,035 *** | 530 | 2,289 *** | 618 |
| Mental outpatient hospital care^b^ | 1,977 *** | 398 | 1,934 *** | 406 | 1,787 *** | 387 |
| Mental inpatient hospital care^b^ | 323 | 1,415 | 825 | 1,683 | 596 | 1,326 |
| Somatic outpatient hospital care^b^ | 269 | 376 | 264 | 368 | 156 | 287 |
| Somatic inpatient hospital care^b^ | -489 | 606 | -302 | 571 | -547 | 505 |

Note: The table contains estimated coefficients (in €) of the association between mental health problems at age 5-7 years and health care costs in the following eleven year-period (2006 to 2016). The number of observations N=3,306.

^a^ Marginal effect (ME) and robust standard error (SE) estimated with a GLM model.

^b^ Marginal effect (ME) and robust standard error (SE) estimated with a two-part model combining a logit and a GLM model.

^c^ Gender, first-born child in the family, born small-for-gestational-age or with low-birth-weight, parents are second generation immigrants, child did not live with both biological parents 1st January 2006.

^d^ Mother and/or father had contact to mental health hospital services between 1995 and 2005, mother’s and/or father’s highest education on 1st January 2006 was primary school (up to 9 years schooling), mother and/or father was unemployed in 2005, mother and/or father was among the parents of the full CCC2000 cohort with the 25% highest health care cost between 2000 and 2005.

*p-level <0.1 **p-level <0.05 ***p-level <0.01.
