# Supplementary material for: Evaluation of a screening algorithm using the Strengths and Difficulties Questionnaire to identify children with mental health problems: A five-year register-based follow-up on school performance and healthcare use
Source: PLoS One. 2019 Oct 23;14(10):e0223314. doi: 10.1371/journal.pone.0223314 (PMC6808306; doi:10.1371/journal.pone.0223314)
Supplement: Attachment — Submitted filename: Review_SDQ_PlosOne_2019.docx [file pone.0223314.s002.docx]

The work entitled ***“Evaluation of a screening algorithm using the Strengths and Difficulties Questionnaire to identify children with mental health problems: A five-year register-based follow-up on school performance and healthcare use***" is of great interest. The research is very stimulating; it contains new scientific knowledge and provides comprehensive information for further development of this productive line of research. The data are presented in a clear and easy-to-understand fashion, and the paper is, principally, well-argued and clearly worthy of publication. However, I have some comments to make that should be addressed before I recommend this manuscript for publication

From my point of the view, the introduction would benefit from the inclusion of more literature about the SDQ use in mental health screening worldwide. Considering that the study is about the mental health problems and the parents’ report about mental health, some other studies about this would be advisable. For instance: Bach et al., 2019; Navarro et al., 2019; Ortuño-Sierra, Aritio-Solana, and Fonseca-Pedrero, 2018.

In the results section, it is not clear to me why the authors say that: “To investigate whether this subgroup of children with MHP have an unmet need for care, we use the children’s later school performance as an indicator of the consequences of their MHP at the age of 11-12 years”. In my opinion, this should be at least explained in the introduction. Is it there more literature confirming that a decrease in school performance can be used as an indicator of a increase in mental health problems

In the discussion section authors state that: “The 77.3% of children with MHP who do not receive any SMHC during the follow-up period also obtain a statistically significantly worse GPA at their 9th grade exams than children with no MHP.” I do not clearly understand if the comparison should be between those who have MHP and those without MHP. It is not clear if the difference is because the lack of intervention or because of the fact that they have MHP. Authors explain this, but it seems obvious, so do not really see the point of including this.
